# Development and Validation of the Health Belief Model‐Based Occupational Exposure Risk Perception Scale for Nursing Staff

**DOI:** 10.1155/jonm/4322052

**Published:** 2026-08-19

**Authors:** Lingyun Tian, Qi Qin, Mengyuan Liu, Xinyu Feng, Zhenhuan Cao, Yinglan Li, Jing Jiang

**Affiliations:** ^1^ Department of Health Management Center, The First Affiliated Hospital of USTC, Division of Life Sciences and Medicine, University of Science and Technology of China, Hefei, Anhui, China, ustc.edu.cn; ^2^ Department of Nursing, The First Affiliated Hospital of USTC, Division of Life Sciences and Medicine, University of Science and Technology of China, Hefei, Anhui, China, ustc.edu.cn; ^3^ Institute of Public Health Sciences, Division of Life Sciences and Medicine, University of Science and Technology of China, Hefei, China, ustc.edu.cn; ^4^ School of Nursing, Bengbu Medical University, Bengbu, Anhui, China, bbmc.edu.cn; ^5^ Xiangya School of Nursing, Central South University, Changsha, Hunan, China, csu.edu.cn; ^6^ School of Nursing, The Hong Kong Polytechnic University, Hong Kong, China, polyu.edu.hk; ^7^ Teaching and Research Section of Clinical Nursing, Xiangya Hospital of Central South University, Changsha, Hunan, China, csu.edu.cn; ^8^ National Clinical Research Center of Geriatric Disorder, Xiangya Hospital of Central South University, Changsha, Hunan, China, csu.edu.cn; ^9^ Henan Provincial Chest Hospital, Zhengzhou University, Zhengzhou, Henan, China, zzu.edu.cn

**Keywords:** Health Belief Model, nursing staff, occupational exposure, risk perception, scale development

## Abstract

**Background:**

Nursing staff frequently encounter serious occupational exposure risks that may adversely affect both their physical well‐being and mental health. Their awareness of these risks plays a pivotal role in shaping protective behaviors and influencing how they recognize and respond to workplace hazards. However, existing assessment tools have several limitations, including a lack of robust theoretical foundation and an inability to reflect the complexity of contemporary clinical environments.

**Methods:**

This multiphase study developed and validated a scale based on the Health Belief Model (HBM). The process included a structured literature review for item generation, a two‐round Delphi consultation, a pilot survey (400 nurses) with exploratory factor analysis (EFA), and a cross‐sectional validation sample (1250 nurses) using confirmatory factor analysis (CFA). Reliability was assessed using Cronbach’s alpha and the Spearman–Brown split‐half coefficient.

**Results:**

The final scale comprised 32 items organized into five theoretically grounded dimensions: perceived susceptibility, perceived severity, perceived benefits, perceived barriers, and self‐efficacy. The results of EFA and CFA supported the proposed five‐factor model. Fit indices suggested an acceptable model fit: chi‐square/df = 2.941; RMSEA = 0.039; CFI = 0.974; NFI = 0.961; IFI = 0.974. The overall Cronbach’s alpha was 0.927. The total Spearman–Brown split‐half coefficient was 0.659, and dimension‐level coefficients ranged from 0.832 to 0.937.

**Conclusion:**

The proposed HBM‐based Occupational Exposure Risk Perception Scale (HBM‐OERPS) showed initial validity evidence and good internal consistency for assessing nurses′ perceptions of occupational hazards within an HBM framework.

**Nursing Implications:**

The HBM‐OERPS provides an effective tool for future assessment of occupational exposure risk beliefs among nursing staff. This approach assists in identifying domain‐specific risk‐belief deficits and guides the design of targeted occupational safety education, resource allocation, and management strategies.

## 1. Introduction

Nurses face a wide array of occupational hazards that pose significant threats to both their physical well‐being and psychological health [1]. These risks encompass multiple domains: Biological hazards caused by blood‐borne pathogens [2], chemical hazards caused by disinfectants and antitumor drugs [3, 4], physical injuries such as needlestick or sharps injuries [5], ergonomic hazards leading to musculoskeletal diseases [6], and psychosocial risks such as workplace violence [7]. Survey data from China revealed that 81.37% of frontline nurses experienced at least one work‐related injury within the past year [8], underscoring an urgent need for robust, evidence‐informed prevention strategies.

Risk perception, defined as an individual’s subjective assessment of the nature and seriousness of potential hazards [9, 10], serves as a critical cognitive driver in shaping protective responses. For nurses, how they appraise occupational exposure risks significantly influences their adherence to safety guidelines, consistent use of personal protective equipment, and willingness to report exposure events [11]. A heightened awareness of such risks tends to prompt proactive measures aimed at minimizing harm [12]. Conversely, underestimating these risks may foster a false sense of security and result in suboptimal protective practices, while overestimating can trigger excessive worry and impair clinical decision‐making [10].

Several assessment tools have been designed to evaluate occupational risk awareness among nurses. Zhang et al. [13] developed a 28‐item questionnaire across six domains, demonstrating strong reliability (Cronbach’s alpha = 0.947). Although this scale effectively describes general occupational risk awareness, it does not systematically differentiate the underlying cognitive or motivational factors that shape nurses’ decisions to engage in, or refrain from, protective practices. Li et al. [14] constructed the Occupational Hazard Risk Cognition Scale based on the classification system of occupational hazards in the medical industry and tested its reliability and content validity. However, its generalizability across healthcare settings and geographical areas remains to be empirically confirmed. The Occupational Exposure Risk Awareness Scale (OERAS) developed by Mu and Qian [15] demonstrated good psychometric properties (Cronbach’s alpha = 0.85; CVI = 0.88). Nevertheless, this scale was specifically tailored for staff working in disinfection supply centers and may not capture the broader and more complex risk perception patterns of general nursing staff. Existing tools fail to adequately capture nurses′ perceptions of the likelihood and severity of occupational exposure, their appraisal of the benefits and barriers of protective measures, and their confidence in performing occupational protection behaviors. Given these limitations, a belief‐based instrument is needed to evaluate nurses′ occupational exposure risk perception.

The Health Belief Model (HBM) is a theoretical framework for explaining and predicting health‐related behaviors. The model posits that an individual’s protective behavior is driven by their perceptions of health threats, expected benefits, perceived barriers, and confidence in performing recommended actions [16]. Previous studies have applied the HBM to occupational and clinical safety, suggesting that nurses’ risk‐related beliefs, perceived benefits of protection, perceived barriers, and self‐efficacy are relevant to safety‐related behavior [17]. Guided by the HBM’s theoretical structure and the concept of occupational exposure risk perception, the present instrument targeted five latent belief dimensions: perceived susceptibility, perceived severity, perceived benefits, perceived barriers, and self‐efficacy.

To address the limitations in target population coverage, theoretical differentiation of risk beliefs, and psychometric reporting, this study developed and validated the HBM‐based Occupational Exposure Risk Perception Scale (HBM‐OERPS). The specific objectives were: (1) to generate an initial item pool based on HBM dimensions via literature review and expert consultation; (2) to refine the item pool through a two‐round Delphi process and pilot testing; (3) to examine the factor structure using exploratory factor analysis (EFA) and confirmatory factor analysis (CFA); and (4) to evaluate the scale’s content validity, construct validity, convergent validity, discriminant validity, and reliability among nursing staff.

## 2. Methods

### 2.1. Step 1: Development of the HBM‐OERPS

#### 2.1.1. Development of the Item Pool

The initial item pool was derived from the HBM and nurses′ perceptions of occupational exposure risks. Candidate items were organized according to five HBM constructs: perceived susceptibility, perceived severity, perceived benefits, perceived barriers, and self‐efficacy. Items were refined and adapted from validated scales and relevant empirical studies to enhance conceptual alignment, linguistic clarity, and contextual appropriateness for the nursing population.

A structured literature review was conducted in Chinese and international academic databases to identify studies published between January 1, 2014, and May 1, 2024. Chinese‐language sources included Wanfang Data, CNKI, SinoMed, and VIP; English‐language sources included Web of Science, PubMed, and Embase. Key search terms comprised occupational exposure, occupational hazard, risk cognition, risk perception, nurses, nursing staff, scales, questionnaires, instruments, and evaluation tools. Database‐specific search strategies were applied and supplemented with backward citation tracking. Eligible studies focused primarily on nurses or nursing personnel and examined occupational exposure risk perception, occupational risk awareness, or relevant measurement tools. Duplicate publications, nonresearch materials, conference proceedings, editorials, commentaries, protocol papers, and studies for which the full text was unavailable were excluded. Two independent reviewers screened titles and abstracts, followed by full‐text assessment. Disagreements were resolved through discussion with a senior reviewer when necessary. The extraction sheet captured information on author, year, country/region, study design, population, sample size, instrument, dimensions, items, and psychometric indicators. The screening summary, characteristics of the included studies, and the mapping of extracted content to HBM‐OERPS domains are provided in Multimedia Appendix 1.

#### 2.1.2. Delphi

A two‐round Delphi technique was employed to refine the item pool and evaluate content validity. Specialists were considered eligible when they fulfilled the following criteria: (1) possessed an intermediate or senior professional title; (2) had been working in their professional domain for more than 10 years, with solid theoretical knowledge, extensive practical experience, and the capacity to provide constructive suggestions; and (3) demonstrated interest in this study and agreed to complete the expert consultation questionnaire. Ultimately, 18 experts from 10 provincial‐level administrative regions across China were involved. Their disciplinary backgrounds encompassed nursing occupational safety, nursing administration, hospital epidemiology and infection prevention, frontline clinical nursing, and nursing education [18].

The first round of the Delphi questionnaire comprised three core components: an overview of the research context, an original item pool, and experts’ demographic information. For item evaluation, experts rated each item’s significance using a 5‐point Likert scale, ranging from *Very Important* (5 points) to *Extremely Unimportant* (1 point). In the subsequent round, experts reassessed both the importance and relevance of the items. The relevance was evaluated with a four‐level Likert scale, ranging from *Highly relevant* (4 points) to *Completely Irrelevant* (1 point). Additionally, experts were invited to provide constructive feedback, such as suggestions for revision, removal, or addition, with the corresponding illustrations. Questionnaires were distributed in the form of e‐mail or the WeChat platform, and the collection deadline was determined based on the specific requirements and complexity of each iteration.

Statistical analyses for the Delphi process were conducted using SPSS 26.0. Items were retained for further evaluation if they met all three criteria: (1) a mean importance rating exceeding 3.50, (2) a proportion of perfect scores above 20%, and (3) a coefficient of variation below 0.25. Expert engagement was gauged by questionnaire response rate. Expert authority was quantified using the authority coefficient (Cr), defined as the average of the familiarity coefficient (Cs) and judgment coefficient (Ca), i.e., Cr = (Cs + Ca)/2, with Cr ≥ 0.70 adopted as the minimum acceptable level [19]. The mean importance score and full‐score rate were used to reflect the concentration of expert opinion, while Kendall’s coefficient of concordance (W) and the coefficient of variation reflected interexpert agreement. Item deletion, revision, merging, and addition decisions were determined by quantitative Delphi indicators and qualitative expert comments.

#### 2.1.3. Pilot Survey, Item Evaluation, and Selection

A pilot survey was carried out in August 2024 to enhance the measurement tool’s clarity and validity. A total of 400 nursing staff from Grade‐A tertiary general hospitals in Hunan Province, China, were selected via convenience sampling. The inclusion criteria were as follows: (1) formal employment at a Grade‐A tertiary general hospital; (2) registered nurses actively engaged in clinical practice; and (3) individuals who provided informed consent and chose to participate voluntarily. Exclusion criteria were as follows: (1) internship nurses, nurses undergoing training, or standardized training nurses; (2) unable to participate due to physical illness or other reasons; and (3) absence from work during the survey period (e.g., maternity leave, sick leave).

Item evaluation was conducted through critical value analysis, Pearson’s correlation coefficient, Cronbach’s alpha estimation, item‐content review, and EFA. In the critical value analysis, respondents were classified into high‐ and low‐scoring groups according to the upper and lower quartiles (27%) of overall scale scores; item‐level performance between the two groups was compared using independent‐samples *t*‐tests, and effect sizes were estimated with Cohen’s d. Pearson’s correlation coefficient was employed to evaluate associations between the overall scale score and each subscale, as well as between individual items and their respective subscales. Cronbach’s alpha was computed for the entire scale and each subscale, and corrected item‐total correlation (CITC) was calculated. EFA was conducted using principal component analysis with varimax rotation, retaining factors with eigenvalues > 1 and a cumulative contribution rate > 50%. The Kaiser–Meyer–Olkin (KMO) statistic and Bartlett’s test of sphericity were used to assess sampling adequacy. Items were removed if they met at least two of the following criteria: (1) nonsignificant item‐dimension correlation (*p* > 0.05); (2) item‐dimension Pearson correlation coefficient < 0.40; (3) CITC < 0.40 coupled with a meaningful increase in Cronbach’s alpha after deletion; and (4) low primary factor loading (< 0.45) or ambiguous cross‐loading (difference between two‐factor loadings < 0.20). Sensitivity analyses were conducted for items with relatively low but still acceptable loadings to determine whether their removal altered explained variance or reliability.

### 2.2. Step 2: Validation of the HBM‐OERPS

The HBM‐OERPS was validated through a cross‐sectional validation study conducted in September 2024, using a multistage sampling method. First, Hunan Province was divided into five geographical regions. From each region, two cities or autonomous prefectures were randomly selected, and one Grade‐A tertiary general hospital was randomly selected from each selected city or prefecture, yielding a total of ten hospitals. Within each hospital, eligible registered nurses from major clinical departments were invited to participate with the assistance of nursing managers. The target sample size was calculated using a formula for estimating a mean, based on the pilot‐survey standard deviation (SD = 17.14), an allowable error of 1.0, alpha = 0.05, and a normal deviate of 1.96, resulting in a minimum sample size of 1108 participants. Accounting for an expected 10%–15% rate of invalid submissions or nonresponses, the desired effective sample size was estimated to fall between 1232 and 1304 participants. Consequently, this study aimed to recruit 1250 respondents. A total of 1351 online questionnaires were distributed through the Wenjuanxing platform, with all questions configured as compulsory to ensure complete responses and eliminate item‐level omissions. Upon completion of data collection, two independent researchers assessed each submitted questionnaire for reliability and consistency. Responses were flagged as potentially invalid and subsequently discarded if they met either of the following conditions: (1) Completion time was under 120 s (significantly shorter than the pilot study’s average response duration of ∼180 s), or (2) over 90% of answers were identical across all items (e.g., uniformly selecting “5” or “1”). Applying these screening criteria led to the removal of 101 submissions, leaving 1250 high‐quality, fully completed questionnaires for analysis, yielding an effective response rate of 92.52%. Notably, the final analytical dataset contained no missing values at the item level. CFA was employed to assess whether the scale structure aligned with the HBM framework. Model fit was evaluated using multiple indices, including chi‐square/df, root mean square error of approximation (RMSEA), goodness‐of‐fit index (GFI), adjusted goodness‐of‐fit index (AGFI), root mean square residual (RMR), comparative fit index (CFI), normed fit index (NFI), and incremental fit index (IFI). Convergent validity was assessed using average variance extracted (AVE) and composite reliability (CR), with AVE > 0.50 and CR > 0.70 indicating acceptable convergent validity. Discriminant validity was deemed acceptable when the square root of the AVE for each construct exceeded its correlations with other constructs. A single‐factor model and a second‐order HBM model were tested as competing models. Multicollinearity among the 32 retained items was examined using variance inflation factor (VIF) and tolerance values.

Content validity was evaluated using the Delphi method, with expert ratings converted into a content validity index (CVI). Acceptable thresholds were set at ≥ 0.78 for item‐level CVI (I‐CVI) and ≥ 0.90 for scale‐level CVI (S‐CVI). Specifically, I‐CVI was computed as the proportion of experts assigning a rating of 3 or 4 (on a 4‐point relevance scale) for each individual item, whereas S‐CVI was derived by calculating the mean of all I‐CVI values across retained items.

In this study, the internal consistency reliability of the entire scale and its five subscales was assessed using Cronbach’s alpha. A threshold of 0.70 or higher was deemed acceptable, while a value of 0.80 or above indicated strong reliability. Additionally, split‐half reliability was calculated using the odd–even division method and the Spearman–Brown correction.

#### 2.2.1. Ethical Considerations

Ethical clearance for this study was granted by the Nursing and Behavioral Medicine Research Ethics Review Committee of Xiangya School of Nursing, Central South University (No. E202415). Before distributing the questionnaire, formal authorization was obtained from the selected hospitals and relevant nursing administrative departments. All participants provided electronic informed consent before completing the questionnaire. To ensure confidentiality and reduce social desirability bias, no personally identifiable information was collected; instead, each respondent was assigned a unique, nontransferable identification code. All collected data were encrypted and stored in a secure, access‐controlled digital repository, with viewing privileges strictly limited to designated research team members. Participants were informed of the study purpose, procedures, voluntary nature, confidentiality measures, and intended use of data and could withdraw from the study at any time without any consequences.

## 3. Results

### 3.1. Development of the Item Pool

A comprehensive literature search yielded 872 records, of which 16 met the inclusion criteria and were selected to inform item development. Among these, six were published in Chinese and ten in English. Geographically, the studies originated from diverse settings: China (6 studies), Turkey (1), the Netherlands (1), Peru (1), the United States (1), Morocco/France (1), India (1), South Korea (1), Nigeria (1), Spain (1), and Brazil (1). Methodologically, fifteen adopted cross‐sectional or psychometric scale development and validation approaches, while one employed qualitative interview‐based inquiry. The synthesized content spanned five key thematic areas: occupational exposure domains, risk perception (or risk cognition), preventive attitudes and behaviors, perceived barriers, and self‐efficacy. Further descriptive information is available in Multimedia Appendix 1.

### 3.2. Delphi

To refine the item pool, Delphi consultation was conducted among 18 experts from 10 regions in China (Beijing, Chongqing, Sichuan, Hunan, Hubei, Fujian, Shandong, Liaoning, Shaanxi, and Xinjiang Uygur Autonomous Region). All experts held at least a bachelor’s degree; approximately 83% possessed a master’s or doctoral qualification; nearly 90% were associate senior or full senior professionals; and about 95% had more than 20 years of work experience. In addition, 72% were master’s or doctoral supervisors, as listed in Table 1.

**TABLE 1:** Expert demographic profile.

Variables	Categories	*n*	%
Gender	Male	4	22.22
	Female	14	77.78

Age (years)	36–45	1	5.56
	46–60	15	83.33
	> 60	2	11.11

Educational level	Doctor	8	44.44
	Master	7	38.89
	Bachelor	3	16.67

Professional title	Senior	15	83.33
	Vice‐senior	2	11.11
	Intermediate	1	5.56

Working years	1–10	1	5.56
	11–20	0	0.00
	21–30	7	38.89
	31–40	7	38.89
	> 40	3	16.66

Tutor status	Master supervisor	9	50.00
	Doctoral supervisor	4	22.22
	No	5	27.78

Areas of expertise	Nursing occupational safety	6	33.33
	Nursing management	11	61.11
	Hospital infection management	4	22.22
	Clinical nursing	9	50.00
	Clinical medicine	1	5.56
	Nursing education	3	16.66

Both consultation rounds achieved a 100% response rate. The authority coefficient was 0.933. The average importance rating for each item exceeded 3.50 in both rounds, and every item attained a full‐score rate above 20%. Across the two rounds, 93.33% and 100% of items had coefficient of variation values below 0.25, respectively. Kendall’s coefficient of concordance (W) was statistically significant in both rounds (Round 1: *W* = 0.255, chi‐square = 201.635, *p* < 0.001; Round 2: *W* = 0.175, chi‐square = 113.546, *p* < 0.001). After Round 1, 22 items were revised, 12 were added, 17 were deleted, and 4 were merged. In Round 2, 10 additional items were refined, resulting in a final 32‐item scale across five domains.

### 3.3. Pilot Survey, Item Evaluation, and Selection

Critical value analysis using independent‐samples *t*‐tests revealed statistically significant differences (*p* < 0.05) between high‐ and low‐scoring groups for all items. Effect‐size estimates from the pilot survey indicated substantial between‐group disparities, with Cohen’s d values ranging from 1.073 to 2.405. For each subscale, the item‐total correlation coefficients were 0.810–0.899 for perceived susceptibility, 0.894–0.951 for perceived severity, 0.931–0.967 for perceived benefits, 0.858–0.925 for perceived barriers, and 0.788–0.906 for self‐efficacy. Correlations between each subscale and the total score ranged from 0.545 to 0.735 (all > 0.400, *p* < 0.05). The overall Cronbach’s alpha was 0.932, while subscale values ranged from 0.909 to 0.974. All items exhibited CITCs > 0.400, and deleting any item did not meaningfully increase Cronbach’s alpha. Accordingly, no item was deleted based on the critical value, correlation, CITC, or Cronbach’s alpha criteria (Table 2).

**TABLE 2:** Results of item evaluation.

Items	Critical value (*t*)	Correlation coefficient (*r*)	CITC	Cronbach’s *α* after removal
A1	−10.749	0.886	0.813	0.880
A2	−11.612	0.899	0.842	0.876
A3	−8.127	0.858	0.761	0.893
A4	−10.790	0.841	0.756	0.893
A5	−9.468	0.810	0.700	0.904
B6	−15.776	0.900	0.858	0.965
B7	−15.368	0.894	0.843	0.967
B8	−16.691	0.944	0.920	0.959
B9	−16.824	0.951	0.927	0.957
B10	−15.523	0.945	0.919	0.958
B11	−16.014	0.935	0.907	0.960
C12	−11.139	0.942	0.911	0.970
C13	−11.312	0.963	0.941	0.965
C14	−11.295	0.961	0.940	0.966
C15	−11.251	0.967	0.948	0.964
C16	−11.961	0.931	0.889	0.974
D17	−13.694	0.858	0.804	0.963
D18	−15.071	0.899	0.862	0.959
D19	−17.610	0.925	0.898	0.956
D20	−17.043	0.925	0.898	0.956
D21	−15.256	0.920	0.890	0.957
D22	−14.486	0.899	0.859	0.959
D23	−15.339	0.925	0.895	0.956
E24	−10.326	0.788	0.728	0.953
E25	−10.020	0.830	0.781	0.950
E26	−8.835	0.828	0.775	0.951
E27	−10.183	0.859	0.821	0.948
E28	−9.231	0.834	0.785	0.950
E29	−9.761	0.899	0.870	0.946
E30	−10.287	0.892	0.862	0.946
E31	−8.645	0.883	0.850	0.947
E32	−9.876	0.906	0.879	0.946

**FIGURE 1:**
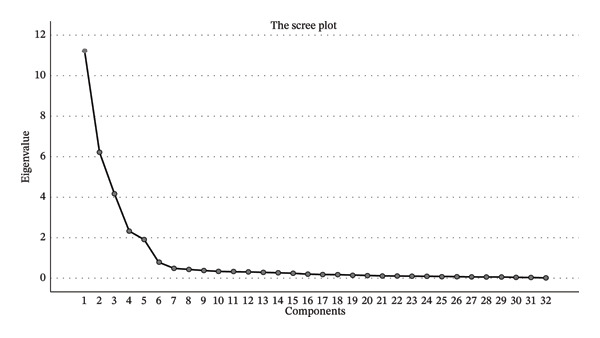
The scree plot.

The initial scale demonstrated excellent sampling adequacy, as indicated by a KMO statistic of 0.921. Bartlett’s test of sphericity was significant (*p* < 0.001), affirming that the dataset met the assumptions required for EFA [20]. Five principal components were identified using varimax rotation, yielding rotated eigenvalues of 6.892, 5.855, 5.086, 4.280, and 3.787, respectively. These values jointly explained 80.94% of the total variance (Table 3). The scree plot also supported the retention of a five‐factor structure, with eigenvalues showing a clear decline after the fifth component (Figure 1).

**TABLE 3:** EFA results.

Factors	Eigenvalue			Extraction sums of squared loadings			Rotation sums of squared loadings		
	Total	Contribution rate (%)	Cumulative contribution rate (%)	Total	Contribution rate (%)	Cumulative contribution rate (%)	Total	Contribution rate (%)	Cumulative contribution rate (%)
1	11.216	35.051	35.051	11.216	35.051	35.051	6.892	21.538	21.538
2	6.232	19.475	54.526	6.232	19.475	54.526	5.855	18.298	39.836
3	4.180	13.062	67.588	4.180	13.062	67.588	5.086	15.895	55.730
4	2.338	7.306	74.895	2.338	7.306	74.895	4.280	13.376	69.107
5	1.935	6.046	80.940	1.935	6.046	80.940	3.787	11.833	80.940

Each item loaded primarily on its designated factor, with loadings ranging from 0.746 to 0.905 (Table 4). Among all items, A5 and E24 showed the lowest loadings, but both remained above 0.70. A supplementary sensitivity analysis based on the pilot‐survey data showed that deleting A5, E24, or both items resulted in negligible changes to Cronbach’s alpha (from 0.932 to 0.932, 0.931, and 0.930, respectively). The corresponding five‐component explained variance changed from 80.940% to 81.687%, 81.713%, and 82.511%, respectively. As both items satisfied the factor‐loading criterion and their deletion did not improve internal consistency, A5 and E24 were retained in the final scale.

**TABLE 4:** Exploratory factor loadings.

Items	Factor loading coefficient				
	Factor 1	Factor 2	Factor 3	Factor 4	Factor 5
A1					0.857
A2					0.866
A3					0.843
A4					0.804
A5					0.755
B6			0.821		
B7			0.837		
B8			0.890		
B9			0.899		
B10			0.898		
B11			0.878		
C12				0.864	
C13				0.893	
C14				0.887	
C15				0.884	
C16				0.847	
D17		0.824			
D18		0.868			
D19		0.901			
D20		0.901			
D21		0.901			
D22		0.885			
D23		0.905			
E24	0.746				
E25	0.788				
E26	0.815				
E27	0.832				
E28	0.820				
E29	0.878				
E30	0.867				
E31	0.875				
E32	0.887				

### 3.4. Validity and Reliability of the HBM‐OERPS

**FIGURE 2:**
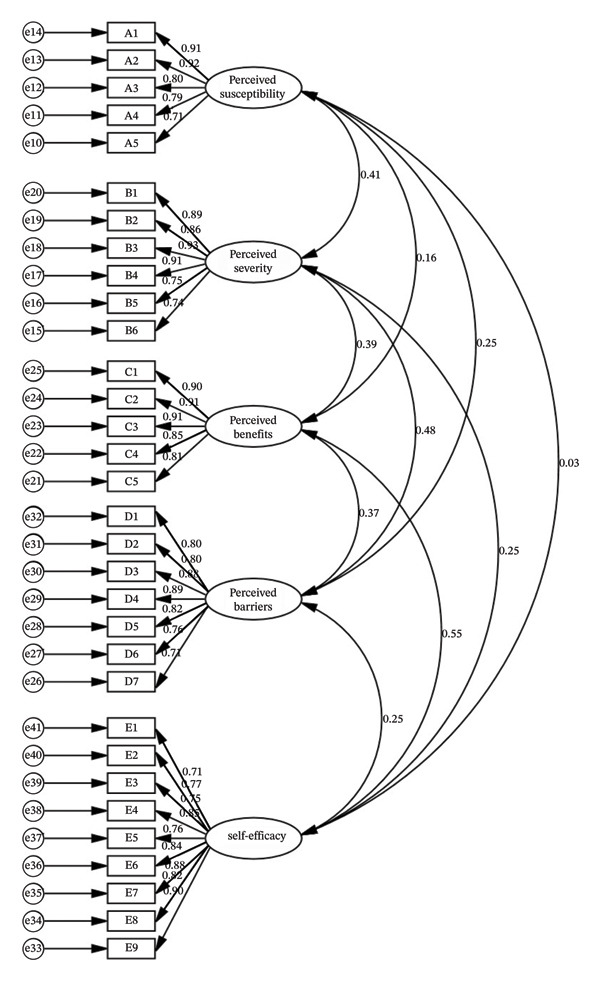
Structural equation modeling for confirmatory factor analysis.

Based on the EFA results, CFA was performed to test the hypothesized factor structure of the OERPS for nursing staff. Using AMOS 26.0, a standardized structural equation model was estimated from 1250 nursing professionals. Prior to conducting CFA, distributional diagnostics were performed. Univariate skewness ranged from −1.831 to −0.297, and kurtosis ranged from −0.962 to 3.949. Multivariate normality was further evaluated using Mardia’s coefficients: skewness = 123.570 and kurtosis = 1426.010 (*z* = 128.093), indicating moderate deviation from multivariate normality. The hypothesized five‐factor model demonstrated adequate fit: chi‐square/df = 2.941, GFI = 0.933, AGFI = 0.922, RMSEA = 0.039, RMR = 0.024, CFI = 0.974, NFI = 0.961, and IFI = 0.974 (Figure 2). Comparisons against competing models further supported this solution: The one‐factor model yielded poor fit (chi‐square/df = 47.243, CFI = 0.364, NFI = 0.359, RMSEA = 0.192), while the second‐order HBM model showed adequate but slightly inferior fit (chi‐square/df = 3.503, CFI = 0.966, NFI = 0.953, RMSEA = 0.045). Multicollinearity diagnostics revealed VIF values ranging from 1.987 to 5.278 and tolerance values from 0.189 to 0.503; only one item exhibited a VIF marginally exceeding the conventional threshold of 5.0. All standardized factor loadings surpassed 0.70, with AVE ranging from 0.656 to 0.769 and CR from 0.916 to 0.945 (Table 5). Discriminant validity was supported, as the square roots of the AVEs exceeded the corresponding interconstruct correlations (Table 6). Content validity indices were as follows: S‐CVI = 0.986 and I‐CVIs ranged from 0.889 to 1.000 (see Table 7).

**TABLE 5:** Results of convergent analysis.

Paths	Estimate	CR	AVE
A5 <‐‐‐ A	0.707	0.916	0.688
A4 <‐‐‐ A	0.787		
A3 <‐‐‐ A	0.796		
A2 <‐‐‐ A	0.925		
A1 <‐‐‐ A	0.912		

B11 <‐‐‐ B	0.738	0.939	0.720
B10 <‐‐‐ B	0.747		
B9 <‐‐‐ B	0.914		
B8 <‐‐‐ B	0.926		
B7 <‐‐‐ B	0.859		
B6 <‐‐‐ B	0.887		

C16 <‐‐‐ C	0.812	0.943	0.769
C15 <‐‐‐ C	0.845		
C14 <‐‐‐ C	0.913		
C13 <‐‐‐ C	0.910		
C12 <‐‐‐ C	0.901		

D23 <‐‐‐ D	0.713	0.930	0.656
D22 <‐‐‐ D	0.762		
D21 <‐‐‐ D	0.816		
D20 <‐‐‐ D	0.893		
D19 <‐‐‐ D	0.877		
D18 <‐‐‐ D	0.799		
D17 <‐‐‐ D	0.797		

E32 <‐‐‐ E	0.896	0.945	0.659
E31 <‐‐‐ E	0.823		
E30 <‐‐‐ E	0.882		
E29 <‐‐‐ E	0.841		
E28 <‐‐‐ E	0.758		
E27 <‐‐‐ E	0.852		
E26 <‐‐‐ E	0.747		
E25 <‐‐‐ E	0.770		
E24 <‐‐‐ E	0.714		

**TABLE 6:** Results of discriminant analysis.

	A	B	C	D	E
A	0.688				
B	0.414	0.720			
C	0.160	0.391	0.769		
D	0.249	0.479	0.370	0.656	
E	0.027	0.247	0.553	0.253	0.659
Square root of AVE	0.830	0.849	0.877	0.810	0.812

*Note:* The values on the diagonal are the AVE values of each dimension.

**TABLE 7:** Results of content validity.

Item	Number of experts rating 3 or 4	I‐CVI
A1	18	1.000
A2	18	1.000
A3	18	1.000
A4	17	0.944
A5	18	1.000
B6	18	1.000
B7	17	0.944
B8	18	1.000
B9	16	0.889
B10	17	0.944
B11	18	1.000
C12	18	1.000
C13	18	1.000
C14	18	1.000
C15	18	1.000
C16	17	0.944
D17	18	1.000
D18	18	1.000
D19	18	1.000
D20	18	1.000
D21	17	0.944
D22	18	1.000
D23	18	1.000
E24	18	1.000
E25	18	1.000
E26	18	1.000
E27	18	1.000
E28	18	1.000
E29	18	1.000
E30	17	0.944
E31	18	1.000
E32	18	1.000

The overall Cronbach’s alpha was 0.927. Cronbach’s alpha coefficients for the subscales of perceived susceptibility, perceived severity, perceived benefits, perceived barriers, and self‐efficacy were 0.915, 0.935, 0.943, 0.929, and 0.943, respectively. The Spearman–Brown split‐half coefficient was 0.659 for the total scale. Dimension‐level split‐half coefficients for subscales were 0.832, 0.937, 0.884, 0.891, and 0.911, respectively. The final version of the OERPS for nursing staff is presented in Multimedia Appendix 2.

## 4. Discussion

### 4.1. Principal Findings

This study developed and initially validated the HBM‐OERPS for nursing staff. The final 32‐item scale covered five HBM dimensions: perceived susceptibility, perceived severity, perceived benefits, perceived barriers, and self‐efficacy. Both EFA and CFA supported the proposed five‐factor structure. The scale demonstrated satisfactory content validity, convergent validity, discriminant validity, and internal consistency. Unlike existing occupational risk assessment instruments [13–15], the HBM‐OERPS explicitly integrates modifiable belief constructs grounded in health belief theory and simultaneously addresses perceived exposure threats and action‐related beliefs. These features provide a clearer basis for characterizing nurses′ occupational exposure risk perception and identifying domains relevant to occupational safety education and management.

#### 4.1.1. Step 1: Development of the HBM‐OERPS

The development of HBM‐OERPS is based on the HBM, and the concept of health behavior is determined by five aspects: perceived susceptibility, perceived severity, perceived benefit, perceived barriers, and self‐efficacy. This conceptual framework guides the development of the instrument, enabling it to comprehensively capture multiple dimensions of nurses′ perceived occupational exposure risks. To guarantee the instrument’s relevance, clarity, and comprehensiveness, the study integrates insights from an extensive literature review and targeted consultations with domain experts.

Within the HBM, perceived vulnerability and perceived severity jointly constitute the component of threat perception, which affects individuals′ awareness and attention to potential health risks [21]. For perceived susceptibility, various occupational hazards in the clinical environment were checked. These hazards include physical exposure, sharp instrument injury, noise, radiation, and ultraviolet rays (A1); biological exposure of patients′ blood, body fluids, secretions, and contaminated instruments that may lead to bacterial, viral, or fungal infection (A2); chemical exposure of toxic or irritating drugs, disinfectants, gases, and particulate matter (A3); musculoskeletal injuries, varicose veins, and sprains of lower limbs caused by professional sports (A4); and psychosocial exposure, workplace violence, and occupational stress (A5). These items are extremely important for capturing the common risks perceived by nurses in the working environment and make up for the limitation of previous tools that only focus on a single occupational hazard category. As far as the perceived severity is concerned, the nurses’ views on the consequences of occupational exposure were evaluated. Specifically, it examined factors across multiple domains: physical well‐being (B6), financial stability (B7), mental health, such as anxiety and depression (B8), fulfilling other social roles (family responsibilities) (B9), work efficiency and quality (B10), and long‐term occupational health outcomes (B11). These items reflect the various effects of occupational exposure, and its adverse effects go far beyond direct physical injury, extending to all aspects of the nurses′ personal and professional lives.

Perceived benefits and perceived barriers belong to the behavior evaluation part of HBM, which reflects the personal evaluation of recommended protective measures [22]. For the perceived benefits, we checked nurses’ beliefs in the effectiveness of protective measures to reduce specific occupational risks. The detailed items included the following: safe injection practice to prevent sharp injuries (C12); proper use of personal protective equipment to reduce radiation, ultraviolet radiation, and toxic chemicals (C13); standard preventive measures and hand hygiene to reduce the infection of pathogenic microorganisms (C14); correct working posture to prevent musculoskeletal diseases (C15); and actively seek psychological support and learn stress management skills to relieve occupational stress (C16). These factors are very important for understanding the motivation of nurses to participate in protective behaviors, as the HBM believes that individuals are more likely to adopt recommended actions when they perceive such behaviors as advantageous. As far as perceived barriers are concerned, the factors that cause nurses to fail to implement protective measures are evaluated. These factors include an imperfect management system and lack of legal protection (D17), heavy workload (D18), a lack of self‐protection awareness (D19), a lack of knowledge and skills of occupational protection (D20), a lack of protective equipment (D21), a poor safety culture of departments (D22), and a lack of feedback on protective effects (D23). These initiatives address practical challenges nurses face in clinical settings, highlighting a critical gap: Despite recognizing the advantages of protective practices, nurses often fail to implement them due to contextual barriers. Given the demanding nature of nursing roles and the significant impact of organizational constraints on safety‐related behaviors, a comprehensive assessment of such obstacles is essential.

Self‐efficacy, the fifth factor of HBM, refers to an individual’s confidence in their ability to successfully carry out the recommended health‐protective actions [23]. On this basis, we initially assessed nurses’ confidence in taking measures to reduce occupational exposure (E24). Subsequently, we established a set of essential protective measures to be consistently implemented in clinical settings, including: adhering to safe injection protocols in daily clinical practice (E25); proper use of personal protective equipment (E26); taking standard precautions and maintain hand hygiene (E27); adopting a healthy working posture conforming to ergonomic principles (E28); participating in occupational protection training to improve awareness, knowledge, and skills (E29); actively reporting the risk factors and accidents found in the workplace (E30); participating in activities to improve occupational environmental safety (E31); and evaluation of cooperation protection effectiveness (E32). These settings stem from the recognition that self‐efficacy has behavioral specificity, and nurses′ confidence varies depending on different protective measures. Extensive empirical evidence consistently identifies self‐efficacy as a robust determinant of both initiating and sustaining health‐promoting behaviors [24, 25]. By integrating self‐efficacy assessments into the HBM‐OERPS, the evaluation yields targeted insights into precisely where nurses require enhanced support, skill development, or resource allocation, thus improving nurses’ confidence and ability in occupational safety practice. This focus holds special significance in nursing practice, given the complexity and variability of clinical settings, which necessitate the application of diverse protective strategies tailored to specific contexts.

The item‐development process demonstrated that the HBM can effectively organize a broad spectrum of occupational exposure content into coherent, theory‐driven domains. The finalized scale encompasses both prevalent clinical exposure hazards and belief‐related constructs, thereby extending existing assessment instruments that often target narrower populations or lack explicit theoretical differentiation. At this stage, the primary contribution of the HBM‐OERPS is measurement‐oriented: It provides a structured framework for characterizing nurses’ risk beliefs across five core dimensions: perceived susceptibility, perceived severity, perceived benefits, perceived barriers, and self‐efficacy. Cues to action were not retained as an independent latent dimension, as they typically represent external prompts or situational triggers rather than stable individual perceptions. In this study, action‐related contexts were embedded within item content reflecting training participation, risk reporting, feedback, and safety improvement activities.

#### 4.1.2. Step 2: Validation of the HBM‐OERPS

The structural validity of the HBM‐OERPS was examined using EFA and CFA. EFA revealed a clear five‐factor structure, with all items loading acceptably on their theoretical constructs and no ambiguous cross‐loadings. CFA further supported the hypothesized structure, with RMSEA, RMR, CFI, NFI, and IFI indicating good fit. Although the chi‐square/df ratio of 2.941 exceeded the more stringent threshold of 2.0, chi‐square‐based statistics are known to be sensitive to large sample sizes. Therefore, model evaluation relied on multiple fit indices rather than a single statistic. Convergent and discriminant validity were supported by AVE, CR, and the Fornell–Larcker criterion. Internal consistency was strong overall, as reflected by high Cronbach’s alpha coefficients; however, the total split‐half reliability fell below the conventional 0.70 benchmark. Notably, subscale‐level split‐half values remained acceptable, suggesting that the lower global coefficient likely reflects the multidimensionality and conceptual heterogeneity across HBM constructs rather than poor reliability within individual dimensions. Nevertheless, future research should examine test–retest reliability and consider item refinement or alternative splitting methods.

The HBM‐OERPS demonstrates promising reliability and validity as an instrument for assessing nurses’ perceptions of occupational exposure risks. Nurses from different clinical departments of Grade‐A tertiary general hospitals participated, ensuring that the tool was grounded in real‐world clinical experience. Grounded in the HBM, this instrument offers a systematic framework for examining nurses′ perceptions of exposure hazards, appraisal of potential health consequences, assessment of preventive strategies, recognition of implementation barriers, and self‐efficacy in adopting protective behaviors. For nursing administrators, the scale can help identify gaps in occupational safety training or deficiencies in resource support. For researchers, it provides a conceptual foundation for investigating associations among risk perception, protective behaviors, safety culture, workplace conditions, ethical practice, and occupational health outcomes.

### 4.2. Limitations, Strengths, and Future Directions

This study has several limitations. First, the sample was drawn exclusively from Grade‐A tertiary general hospitals in Hunan Province, which may limit generalizability to other regions, healthcare levels, and non‐Chinese settings. Future studies should assess measurement invariance and external validity across diverse populations. Second, the Delphi panel mainly included highly experienced experts, with limited representation of early‐career clinical nurses. In addition, although Kendall’s W in the second Delphi round reached statistical significance, its modest value suggests that expert consensus should be interpreted with caution. Third, the total split‐half reliability coefficient was 0.659, slightly below the conventional threshold of 0.70. This likely reflects the scale’s multidimensional structure and the limitations of the odd–even splitting method, as subscale‐level coefficients were acceptable; nonetheless, further reliability testing and targeted item refinement are warranted. Fourth, test–retest reliability and criterion‐related validity were not assessed; therefore, the scale’s temporal stability and agreement with external criteria require further examination.

Despite these limitations, this study provides a theory‐based instrument for assessing nurses′ occupational exposure risk perception. The HBM‐OERPS may help identify belief patterns and inform educational or organizational support strategies in similar tertiary hospital settings. Future research should focus on cross‐cultural adaptation, broader validation across diverse healthcare settings and populations, and the application of the HBM‐OERPS to guide occupational safety interventions and evaluate their practical effectiveness.

## 5. Conclusions

In this study, the HBM‐OERPS was developed and initially validated as a multidimensional psychometric tool for assessing occupational exposure risk perception among nursing staff. The final scale comprises 32 items covering five core dimensions: perceived susceptibility, perceived severity, perceived benefits, perceived barriers, and self‐efficacy. Psychometric analyses provided evidence for content validity, construct validity, convergent validity, discriminant validity, and internal consistency. Within Chinese tertiary hospitals, the HBM‐OERPS provides a structured, theory‐based instrument for measuring nurses’ occupational exposure risk perception within the HBM framework. Further validation in other healthcare settings and cultural contexts is needed prior to broader application.

## Author Contributions

Lingyun Tian: writing–original draft, writing–review and editing, software, methodology, data curation, investigation, and conceptualization. Qi Qin: writing–review and editing, methodology, data curation, and software. Mengyuan Liu: writing–review and editing, methodology, and investigation. Xinyu Feng: writing–review and editing, methodology, and investigation. Zhenhuan Cao: writing–review and editing and data curation. Yinglan Li: writing–review and editing, methodology, investigation, conceptualization, and project administration. Jing Jiang: writing–review and editing, methodology, data curation, software, conceptualization, and project administration.

## Funding

This study was supported by grants from the Youth Fund Project of the National Natural Science Foundation of China (Grant No. 72304261).

## Disclosure

All authors have read and agreed to the final version of the manuscript. This manuscript follows the STROBE checklist for cross‐sectional studies.

## Ethics Statement

This study was approved by the Nursing and Behavioral Medicine Research Ethics Review Committee of Xiangya School of Nursing, Central South University (Approval No. E202415). The study was conducted in compliance with the ethical principles of the Declaration of Helsinki. Informed consent was obtained from all individual participants included in the study.

## Conflicts of Interest

The authors declare no conflicts of interest.

## Supporting Information

Additional supporting information can be found online in the Supporting Information section.

## Supporting information


**Supporting Information 1** Multimedia Appendix 1: Supporting Methods for Item Generation and Delphi Consultation.

**Supporting Information 2** Multimedia Appendix 2: Final HBM‐OERPS Scale.

**Supporting Information 3** STROBE_checklist_cross‐sectional.

## Data Availability

The datasets analyzed during this study are available from the corresponding author upon reasonable request.

## References

[1] Medeni İ. , Alagüney M. E. , and Medeni V. , Occupational Injuries Among Healthcare Workers: a Nationwide Study in Turkey, Frontiers in Public Health. (2024) 12, no. 2024, 10.3389/fpubh.2024.1505331. PMC11655324 39703481

[2] Rasmussen P. U. , Uhrbrand K. , Frederiksen M. W. , and Madsen A. M. , Work in Nursing Homes and Occupational Exposure to Endotoxin and Bacterial and Fungal Species, Annals of Work Exposures and Health. (2023) 67, no. 7, 831–846, 10.1093/annweh/wxad032. 37300561 PMC10410494

[3] Leso V. , Sottani C. , Santocono C. , Russo F. , Grignani E. , and Iavicoli I. , Exposure to Antineoplastic Drugs in Occupational Settings: a Systematic Review of Biological Monitoring Data, International Journal of Environmental Research and Public Health. (2022) 19, no. 6, 10.3390/ijerph19063737. PMC8952240 35329423

[4] Wang Y.-X. , Dumas O. , Varraso R. et al., Occupational Exposure to Disinfectants and Risk of Incident Cardiovascular Disease Among US Nurses: the Nurses’ Health Study II, Environmental Health Perspectives. (2025) 133, no. 5, 10.1289/EHP14945. PMC12063792 40163383

[5] Abdelmalik M. A. , Alhowaymel F. M. , Fadlalmola H. et al., Global Prevalence of Needle Stick Injuries Among Nurses: a Comprehensive Systematic Review and Meta-Analysis, Journal of Clinical Nursing. (2023) 32, no. 17-18, 5619–5631, 10.1111/jocn.16661. 36841963

[6] Gülnar E. , Bıyık Bayram Ş. , Çökelek F. , Tikit Ö. , and Çalışkan N. , Ergonomic Risks and Musculoskeletal Disorders: a Descriptive Study on Nurses, Work. (2026) 84, no. 3, 711–721, 10.1177/10519815261422189. 41712482

[7] Kumari A. , Sarkar S. , Ranjan P. et al., Interventions for Workplace Violence Against Health-Care Professionals: a Systematic Review, Work. (2022) 73, no. 2, 415–427, 10.3233/WOR-210046. 35431213

[8] Zhang H. , Chen W. , Liu Y. , Shu Y. , and Jin H. , A Retrospective Analysis of 684 Cases of Occupational Exposure Among Healthcare Workers in a General Hospital, Journal of Third Military Medical University. (2018) 40, no. 3, 264–269, 10.16016/j.1000-5404.201709081.

[9] Xie X. F. and Xu L. C. , An Overview and Theoretical Framework of Risk Perception Research, Psychological Dynamics. (1995) 3, no. 2, 17–22.

[10] Shao X. Q. , Risk Perception Characteristics and Influencing Factors of Nurses in Military Hospitals Master’s Thesis, 2024, Naval Medical University of the Chinese People’s Liberation Army, 10.26998/d.cnki.gjuyu.2024.000149.

[11] Al-Dossary R. N. , AlMahmoud S. , Banakhar M. A. et al., The Relationship Between Nurses’ Risk Assessment and Management, Fear Perception, and Mental Wellbeing During the COVID-19 Pandemic in Saudi Arabia, Frontiers in Public Health. (2022) 10, 10.3389/fpubh.2022.992466. PMC9685659 36438216

[12] Pirot C. , Benoist H. , and Saint-Lorant G. , Impact of Lack of Knowledge on Risk Perception and Protective Practices of Home Nurses Handling Antineoplastic Drugs, Journal of Oncology Pharmacy Practice. (2024) 30, no. 2, 313–321, 10.1177/10781552231174181. 37151100

[13] Zhang X. , Development of a Risk Perception Questionnaire for Nursing Staff and a Study on Its Influencing Factors Master’s Thesis, 2016, Fourth Military Medical University.

[14] Li H. , Song Y. N. , and Wang X. F. , Development and Evaluation of a Nursing Occupational Risk Assessment Tool, Chinese Journal of Nursing. (2008) 43, no. 7, 651–654, 10.3761/j.issn.0254-1769.2008.07.030.

[15] Mu Y. and Qian X. , Reliability and Validity Testing of the Occupational Exposure Risk Awareness Scale (OERAS) for Medical Personnel in Disinfection Supply Centers, Risk Management and Healthcare Policy. (2025) 18, 3911–3919, 10.2147/RMHP.S558345. 41445940 PMC12724234

[16] Janz N. K. and Becker M. H. , The Health Belief Model: a Decade Later, Health Education Quarterly. (1984) 11, no. 1, 1–47, 10.1177/109019818401100101. 6392204

[17] Senarath N. , De Silva D. , Rathnayake R. , Warnakulasuriya S. , Meegoda M. , and Jayasinghe S. S. , Perceptions of Occupational Exposure and Adherence to Safety Measures of Handling Systemic Anti-Cancer Therapy (SACT) Among Oncology Nurses at the National Cancer Institute, Sri Lanka, International Journal of Risk & Safety in Medicine. (2025) 36, no. 4, 248–259, 10.1177/09246479251346172. 40451741

[18] Keeney S. , Hasson F. , and McKenna H. , Consulting the Oracle: Ten Lessons from Using the Delphi Technique in Nursing Research, Journal of Advanced Nursing. (2006) 53, no. 2, 205–212, 10.1111/j.1365-2648.2006.03716.x. 16422719

[19] Chen S. , Cao M. , Zhang J. , Yang L. , Xu X. , and Zhang X. , Development of the Health Literacy Assessment Instrument for Chronic Pain Patients: a Delphi Study, Nursing Open. (2023) 10, no. 4, 2192–2202, 10.1002/nop2.1468. 36564937 PMC10006601

[20] Feng X. , Luo H. , Liu M. et al., Development and Validation of Knowledge, Attitude and Practice Questionnaire for Pediatric Nurses to Prevent Central Venous Device-Related Thrombosis in Hospitalized Children, Nurse Education in Practice. (2023) 71, 10.1016/j.nepr.2023.103694. 37453368

[21] Pakravan-Charvadeh M. R. and Maleknia R. , The Role of Beliefs and Behavioral Intentions in the Analysis of Community Health Responses to Climate Change, Scientific Reports. (2026) 16, no. 1, 10.1038/s41598-026-35106-3. PMC12873159 41507419

[22] Fang L. , Zhang Q. , Zhou N. , Chen J. , and Lou H. , Influencing Factors and Mechanisms Promoting Proactive Health Behavior Intention: an Integration of the Health Belief Model and the Theory of Planned Behavior, Frontiers in Public Health. (2025) 13, 10.3389/fpubh.2025.1629046. PMC12287080 40709034

[23] Park J.-H. , Sherman L. D. , Smith M. L. , Patterson M. S. , and Prochnow T. , The Association Between Health Belief Model Components and Self-Care Practices Among Black/African American Men with Type 2 Diabetes, International Journal of Environmental Research and Public Health. (2025) 22, no. 3, 10.3390/ijerph22030414. PMC11941972 40238475

[24] Wang Y. , Construction and Application of a Self-Management Health Education Program for Ischemic Stroke Patients Based on the Health Belief Model Master’s Thesis, 2024, Henan University, 10.27114/d.cnki.ghnau.2023.000545.

[25] Wang W. Q. and Wang X. F. , The Impact of Empowerment and Self-Efficacy on Self-Management Behaviors in Elderly Patients with Chronic Diseases, Electronic Journal of Practical Clinical Nursing Science. (2020) 5, no. 2.

[26] Boateng G. O. , Neilands T. B. , Frongillo E. A. , Melgar-Quiñonez H. R. , and Young S. L. , Best Practices for Developing and Validating Scales for Health, Social, and Behavioral Research: a Primer, Frontiers in Public Health. (2018) 6, 10.3389/fpubh.2018.00149. PMC6004510 29942800

[27] Copanitsanou P. , Fotos N. , and Brokalaki H. , Effects of Work Environment on Patient and Nurse Outcomes, British Journal of Nursing. (2017) 26, no. 3, 172–176, 10.12968/bjon.2017.26.3.172. 28185485

[28] Dehghani A. , Mosalanejad L. , and Dehghan-Nayeri N. , Factors Affecting Professional Ethics in Nursing Practice in Iran: a Qualitative Study, BMC Medical Ethics. (2015) 16, no. 1, 10.1186/s12910-015-0048-2. PMC4565012 26354119

[29] Lee S. E. and Dahinten V. S. , The Enabling, Enacting, and Elaborating Factors of Safety Culture Associated with Patient Safety: a Multilevel Analysis, Journal of Nursing Scholarship. (2020) 52, no. 5, 544–552, 10.1111/jnu.12585. 32573867

