# Supplementary material for: Development and Validation of the Health Belief Model‐Based Occupational Exposure Risk Perception Scale for Nursing Staff
Source: J Nurs Manag. 2026 Aug 19;2026:4322052. doi: 10.1155/jonm/4322052 (PMC13487696; doi:10.1155/jonm/4322052)
Supplement: Supplementary file 2 [file JONM-2026-4322052-s002.docx]

Multimedia Appendix 1. Supplementary Methods for Item Generation and Delphi Consultation

# A. Literature review and item generation

Table S1. Literature-search scope and screening summary for item generation

| **Element** | **Verified information for item generation** |
| --- | --- |
| Databases searched | CNKI, Wanfang, VIP, SinoMed, PubMed, Web of Science, and Embase. |
| Core search concepts | occupational exposure; occupational risk; risk perception/risk cognition/risk awareness; nurses/nursing staff/healthcare workers; scale/questionnaire/instrument/assessment tool. |
| Main database-search period | 1 January 2014 to 1 May 2024. Earlier sources were retained only when identified through reference tracking or when they provided directly relevant domains/items for item generation. |
| Records initially identified | 872 records. |
| Studies retained for item generation | 16 studies: 6 Chinese-language studies and 10 English-language studies. |
| Records not retained | 856 records were not retained because they did not meet the eligibility criteria, including duplicates, irrelevant topics or populations, non-measurement studies, non-research publications, and unavailable or incomplete full text. |
| Use of quality appraisal | Sources were evaluated for relevance to item generation, clarity of population/design, and availability of domains or items. Because the purpose was to generate scale items rather than pool effects, formal risk-of-bias scoring was not used as an exclusion rule. |
| Exclusion-reason categories | Duplicates; irrelevant topic; irrelevant population; not occupational exposure/risk perception; non-measurement study; non-research publication; unavailable full text or incomplete information. |
| Important note | This literature review was used to identify domains and candidate item content for scale development. It was not intended as a systematic review for pooled effect estimation. |

Table S2. Included studies and extracted contribution for item generation

| **No.** | **Study** | **Country/region** | **Design/participants** | **Measurement tool and reported development basis** | **Contribution to item generation** |
| --- | --- | --- | --- | --- | --- |
| 1 | Wu et al. (2024). Current status and influencing factors of risk perception during internet-based nurse practice | China | Cross-sectional; Internet-based nurses (n=219) | Internet-based Nurse Risk Perception Questionnaire; developed through expert consultation and pilot testing before cross-sectional survey. | Personal safety risk; physical-function risk; occupational risk; psychosocial risk; organizational risk; time risk. |
| 2 | Zhu et al. (2023). Development and validation of an Occupational Exposure Risk Perception Scale for central sterile supply department staff | China | Scale development and cross-sectional validation; Central sterile supply department staff (n=226) | Occupational Exposure Risk Perception Scale; developed through literature review, qualitative interviews, Delphi consultation, pilot survey, and cross-sectional validation. | Likelihood of occupational exposure; severity of consequences; participatory dialogue; behavioral confidence; environmental change. |
| 3 | Liu et al. (2020). Risk perception among frontline healthcare workers during the COVID-19 pandemic | China | Cross-sectional; Frontline healthcare workers (n=181) | Self-developed occupational-risk perception questionnaire used in a cross-sectional survey during the COVID-19 pandemic. | Personal safety; physical-function risk; occupational exposure risk; psychosocial risk; organizational risk. |
| 4 | Duan et al. (2017). Occupational risk perception among nurses performing nocturnal hemodialysis | China | Cross-sectional; Hemodialysis nurses (n=50) | Nursing Occupational Risk Assessment Questionnaire; developed through literature review, structured interviews, Delphi consultation, pilot survey, and cross-sectional survey. | Accidental hazards; physical hazards; chemical hazards; biological hazards; work-environment hazards. |
| 5 | Zhang et al. (2016). Development of a risk perception questionnaire for nursing staff | China | Scale development and cross-sectional validation; Nursing staff (n=304) | Risk perception questionnaire for nursing staff; developed through literature review, group discussion, semi-structured interviews, Delphi consultation, and cross-sectional validation. | Personal safety; physical-function risk; occupational exposure risk; psychosocial risk; organizational risk; time risk. |
| 6 | Gu et al. (2011). Occupational risk perception among community nurses in Beijing | China | Cross-sectional; Community nurses (n=99) | Self-developed occupational-risk perception questionnaire based on literature review. | Accidental/invasive injury; physical hazards; chemical hazards; biological hazards; ergonomic hazards; psychosocial factors; organizational factors. |
| 7 | Azizoglu et al. (2019). Self-reported environmental health risks of nurses working in hospital surgical units | Turkey | Cross-sectional; Surgical-unit nurses (n=229) | Self-developed environmental health risk questionnaire based on literature review and expert consultation. | Physical, biological, psychological, ergonomic, radiation, and chemical risk factors. |
| 8 | van Gemert-Pijnen et al. (2006). Effectiveness of protocols for preventing occupational exposure to blood and body fluids in Dutch hospitals | Netherlands | Cross-sectional; Healthcare workers (n=70) | Precaution-compliance questionnaire developed from the literature to evaluate protocol use for preventing blood/body-fluid exposure. | Blood/body-fluid exposure history; risk cognition; knowledge of preventive regulations; attitudes; self-reported behavior; self-efficacy; social/organizational support. |
| 9 | Mendez-Maturrano et al. (2023). Occupational risk perception and associated factors among nurses and physicians in Peruvian health facilities | Peru | Cross-sectional; Nurses (n=2,827) and physicians (n=2,101) | Self-developed occupational-risk perception questionnaire used in a large cross-sectional survey of nurses and physicians. | Perceived frequency of contact with highly contagious disease; perceived frequency of exposure to substances that may affect health. |
| 10 | Lee et al. (2013). Risk perception of musculoskeletal injury among critical care nurses | United States | Cross-sectional; Critical care nurses (n=361) | Risk Perception of Musculoskeletal Injury scale (RPMI), a scale for perceived musculoskeletal-injury risk. | Perceived personal risk; perceived risk for others; tasks related to musculoskeletal injury. |
| 11 | Giurgiu et al. (2016). Well-being and occupational risk perception among healthcare workers: a multicenter study in Morocco and France | Morocco/France | Cross-sectional; Healthcare workers (n=2,863) | Self-developed questionnaire used in a multicenter cross-sectional study of healthcare workers. | Work decision latitude; psychological demands; social-support risk. |
| 12 | Anandadurai et al. (2024). Awareness, perception, and practice regarding needle-stick injury and its prevention among healthcare workers | India | Cross-sectional; Healthcare workers (n=400) | Semi-structured questionnaire assessing awareness, perception, and practice regarding needle-stick injury and prevention. | Perceptions/practices related to sharps disposal, needle recapping, and procedures after splash, sharps, or needle-stick injury. |
| 13 | Choi et al. (2018). Infection-control knowledge, attitude, practice, and risk perception of occupational exposure to Zika virus among nursing students in Korea | South Korea | Cross-sectional; Nursing students (n=249) | Self-developed questionnaire based on literature review, assessing infection-control knowledge, attitude, practice, and occupational-exposure risk perception. | Perceived susceptibility to Zika virus infection; fear of infection. |
| 14 | Aluko et al. (2016). Knowledge, attitudes, and perceptions of occupational hazards and safety practices in Nigerian healthcare workers | Nigeria | Cross-sectional; Healthcare workers (n=290) | Self-developed questionnaire assessing occupational hazards, safety practices, knowledge, attitudes, and perceptions. | Needle-stick injury; contact with retroviral patients; radiation exposure; patient/colleague aggression; contact with body fluids; night duty. |
| 15 | Porras-Povedano et al. (2014). Occupational risks perception in professional nursing practitioners at a health care center | Spain | Cross-sectional; Nurses (n=211) | Self-developed occupational-risk perception questionnaire based on literature review. | Exposure to biological materials; handling and moving weight; occupational stress. |
| 16 | Cezar-Vaz et al. (2009). Risk perception in family health work: study with workers in Southern Brazil | Brazil | Qualitative study; Healthcare workers (n=48) | Qualitative interview guide used to identify workers’ perceived occupational risks. | Physical/mental violence; typical work accidents; emotional exhaustion; lack of problem-solving capacity; occupational disease. |

Table S3. Mapping of literature-derived content to HBM-OERPS domains

| **HBM-OERPS domain** | **Examples of literature-derived content used to inform item generation** |
| --- | --- |
| Perceived susceptibility | Physical hazards (sharp injury, noise, radiation, ultraviolet exposure), biological exposure (blood, body fluids, pathogens), chemical exposure (disinfectants, antineoplastic drugs), ergonomic hazards, and psychosocial risks such as workplace violence and occupational stress. |
| Perceived severity | Potential consequences of occupational exposure for physical health, psychological health, work efficiency/quality, economic burden, social/family roles, quality of life, and long-term occupational health. |
| Perceived benefits | Benefits of standard precautions, safe injection practice, hand hygiene, personal protective equipment, ergonomic posture, occupational protection training, stress-management skills, and psychological support. |
| Perceived barriers | Barriers related to workload, lack of equipment, lack of knowledge or skills, insufficient self-protection awareness, imperfect management systems, weak safety culture, and limited feedback on protection effectiveness. |
| Self-efficacy | Confidence in implementing standard precautions, using personal protective equipment, maintaining hand hygiene, reporting occupational exposure hazards/events, participating in training, improving the occupational environment, and evaluating protection effectiveness. |

# B. Delphi consultation and item revision

Table S4. Delphi process summary

| **Consultation round** | **Expert response** | **Item pool before the round** | **Main item-level revisions** | **Outcome for next step** |
| --- | --- | --- | --- | --- |
| Round 1 | 18 experts invited; 18 valid responses (100%). | 5 primary domains and 40 secondary items. | Reworded 22 items; added 12; deleted/replaced 17; consolidated 4 overlapping items. | Revised 32-item draft for the second Delphi round. |
| Round 2 | Same 18 experts; 18 valid responses (100%). | 5 primary domains and 32 secondary items. | Reworded 10 items for clarity; no deletion, addition, or consolidation required. | Final 5-domain, 32-item draft retained for pilot testing. |

Table S5. Supplementary Delphi indicators and item-decision rationale

| **Component** | **Key quantitative indicators** | **Item-decision rationale** |
| --- | --- | --- |
| Prespecified criteria | Mean importance >3.50; full-score rate >20%; coefficient of variation <0.25; expert authority coefficient >=0.70; I-CVI and S-CVI were used for relevance assessment. | Retention, deletion, revision, addition, and consolidation decisions were based on quantitative indicators together with qualitative expert comments, rather than on a single numerical threshold. |
| Round 1 | Expert authority coefficient = 0.933; Kendall's W = 0.255, P < .001; all candidate items met the mean-importance and full-score-rate criteria; 93.33% of items had coefficient-of-variation values <0.25. | Items were revised, added, deleted/replaced, or consolidated mainly because of conceptual overlap, unclear target/reference group, excessive context specificity, or insufficient fit with the target HBM domain. |
| Round 2 | Kendall's W = 0.175, P < .001; all retained items met the mean-importance and full-score-rate criteria; 100% of items had coefficient-of-variation values <0.25; I-CVI = 0.889-1.000 and S-CVI = 0.986. | Only wording revisions were needed; no further deletion, addition, or consolidation was required, and the 5-domain, 32-item scale was finalized for pilot testing. |
