# Supplementary material for: Development and Validation of the Health Belief Model‐Based Occupational Exposure Risk Perception Scale for Nursing Staff
Source: J Nurs Manag. 2026 Aug 19;2026:4322052. doi: 10.1155/jonm/4322052 (PMC13487696; doi:10.1155/jonm/4322052)
Supplement: Supplementary file 3 [file JONM-2026-4322052-s003.docx]

**Multimedia Appendix 2. Final HBM-OERPS Scale**

**Response options: 1 = strongly disagree, 2 = disagree, 3 = neutral, 4 = agree, 5 = strongly agree. The Chinese version is the validated administration version; the English translation is provided for reference.**

**Scoring: Sum or average items within each dimension to calculate subscale scores. The total score is the sum of all 32 items. No reverse scoring is required.**

Item codes follow the manuscript numbering: A1-A5, B6-B11, C12-C16, D17-D23, and E24-E32.

Table S1. Final Health Belief Model-based Occupational Exposure Risk Perception Scale for Nursing Staff

| Item code | **Dimension** | **Chinese validated item** | **English reference translation** | **Score** |
| --- | --- | --- | --- | --- |
| A1 | Perceived susceptibility | 我可能会遭受锐器伤、噪音、辐射和紫外线等物理性职业暴露 | I could encounter various occupational physical risks, including injuries from sharp objects, excessive noise exposure, ionizing radiation, and ultraviolet (UV) radiation. | 1-5 |
| A2 | Perceived susceptibility | 我可能会遭受患者血液、体液、分泌物、排泄物，以及污染器械等所致细菌、病毒和真菌感染的生物性职业暴露 | I could be exposed to biological occupational hazards caused by bacteria, viruses, and fungal infections from patients' blood, body fluids, secretions, excretions, and contaminated instruments. | 1-5 |
| A3 | Perceived susceptibility | 我可能会遭受有毒或刺激性的药物、化学消毒剂、气体和颗粒物等化学性职业暴露 | I could be exposed to chemical occupational hazards such as toxic or irritating medications, chemical disinfectants, gases, and particulate matter. | 1-5 |
| A4 | Perceived susceptibility | 我可能会遭受职业性肌肉骨骼损伤、下肢静脉曲张、扭伤等运动功能性职业暴露 | I could be exposed to ergonomic occupational hazards such as work-related musculoskeletal disorders, lower limb varicose veins, and sprains. | 1-5 |
| A5 | Perceived susceptibility | 我可能会遭受工作场所暴力、职业压力等心理社会性职业暴露 | I could be exposed to psychosocial occupational hazards such as workplace violence and occupational stress. | 1-5 |
| B6 | Perceived severity | 职业暴露会危害我的身体健康 | Occupational exposure will pose risks to harm my physical health. | 1-5 |
| B7 | Perceived severity | 职业暴露会影响我的经济状况 | Occupational exposure will affect my financial stability. | 1-5 |
| B8 | Perceived severity | 职业暴露会影响我的心理健康（如焦虑、抑郁等） | Occupational exposure will impair my mental health (e.g., anxiety, depression). | 1-5 |
| B9 | Perceived severity | 职业暴露会影响我承担其他社会角色（如家庭角色） | Occupational exposure will impair my ability to fulfill other social roles (e.g., family roles). | 1-5 |
| B10 | Perceived severity | 职业暴露会降低我的工作效率和工作质量 | Occupational exposure will reduce my work efficiency and quality. | 1-5 |
| B11 | Perceived severity | 职业暴露会降低我的职业幸福感 | Occupational exposure will reduce my occupational well-being. | 1-5 |
| C12 | Perceived benefits | 采取减少职业暴露的措施（如安全注射行为等）会降低我受到锐器伤的风险 | Taking measures to reduce occupational exposure (e.g., safe injection practices) will lower my risk of sharp instrument injuries. | 1-5 |
| C13 | Perceived benefits | 采取减少职业暴露的措施（如规范使用个人防护用品等）会降低辐射、紫外线及有毒化学物质等给我带来的危害 | The standardized use of personal protective equipment will mitigate health risks associated with radiation, ultraviolet radiation, and hazardous chemical agents. | 1-5 |
| C14 | Perceived benefits | 采取减少职业暴露的措施（如进行标准预防、坚持手卫生等）会降低我感染病原微生物的风险 | Implementing standard precautions and maintaining hand hygiene will reduce infection risk from pathogenic microorganisms. | 1-5 |
| C15 | Perceived benefits | 采取减少职业暴露的措施（如采用正确的工作姿势等）会降低运动功能性职业暴露的发生率 | Adopting correct working postures will reduce the incidence of ergonomic occupational hazards. | 1-5 |
| C16 | Perceived benefits | 采取减少职业暴露的措施（如主动寻求心理援助，学习应对压力技巧等）有助于我减轻职业压力 | Actively seeking psychological assistance, learning stress coping techniques will help me alleviate occupational stress. | 1-5 |
| D17 | Perceived barriers | 缺乏完善的管理制度和足够的法律保护会影响我采取职业防护措施 | The absence of comprehensive management systems and adequate legal protection will affect my adoption of occupational protective measures. | 1-5 |
| D18 | Perceived barriers | 超负荷工作会影响我采取职业防护措施 | Excessive workload will affect my adoption of occupational protective measures. | 1-5 |
| D19 | Perceived barriers | 缺乏自我防护意识会影响我采取职业防护措施 | The absence of self-protection awareness will affect my adoption of occupational protective measures. | 1-5 |
| D20 | Perceived barriers | 缺乏自我防护知识与技能会影响我采取职业防护措施 | The absence of self-protection knowledge and skills will affect my adoption of occupational protective measures. | 1-5 |
| D21 | Perceived barriers | 缺乏充足物资会影响我采取职业防护措施 | Insufficient supplies will affect my adoption of occupational protective measures. | 1-5 |
| D22 | Perceived barriers | 科室安全文化氛围不足会影响我采取职业防护措施 | An inadequate departmental safety culture will affect my adoption of occupational protective measures. | 1-5 |
| D23 | Perceived barriers | 缺乏防护效果评价会影响我采取职业防护措施 | The absence of a protective effectiveness evaluation will affect my adoption of occupational protective measures. | 1-5 |
| E24 | Self-efficacy | 我认为我有能力采取减少职业暴露的措施 | I believe I have the ability to take measures to reduce occupational exposure. | 1-5 |
| E25 | Self-efficacy | 为了减少职业暴露，我能在工作时实施安全注射行为 | To reduce occupational exposure, I can implement safe injection practices during work. | 1-5 |
| E26 | Self-efficacy | 为了减少职业暴露，我能在工作时规范使用个人防护用品 | To reduce occupational exposure, I can use personal protective equipment in a standardized manner during work. | 1-5 |
| E27 | Self-efficacy | 为了减少职业暴露，我能在工作时落实标准预防、坚持手卫生 | To reduce occupational exposure, I can implement standard precautions and maintain hand hygiene during work. | 1-5 |
| E28 | Self-efficacy | 为了减少职业暴露，我能在工作时依据工效学原理采用合理的工作姿势 | To reduce occupational exposure, I can adopt reasonable working postures based on ergonomic principles during work. | 1-5 |
| E29 | Self-efficacy | 为了减少职业暴露，我能积极参与职业防护培训，增强自我防护意识、知识和技能 | To reduce occupational exposure, I can actively participate in occupational protection training to enhance my self-protection awareness, knowledge, and skills. | 1-5 |
| E30 | Self-efficacy | 为了减少职业暴露，我能主动报告工作中发现的危险因素和事件 | To reduce occupational exposure, I can proactively report risk factors and incidents identified during work. | 1-5 |
| E31 | Self-efficacy | 为了减少职业暴露，我能主动参与改善安全职业环境的活动 | To reduce occupational exposure, I can actively participate in activities to improve the occupational safety environment. | 1-5 |
| E32 | Self-efficacy | 为了减少职业暴露，我能配合使用防护效果评价 | To reduce occupational exposure, I can cooperate with the evaluation of protective effectiveness. | 1-5 |
